# Metaphyseal cones in septic revision total knee arthroplasty: A retrospective cohort study with minimum 2‐year follow‐up

**DOI:** 10.1002/jeo2.70944

**Published:** 2026-10-05

**Authors:** Andrea Pasquini, Lucas Martorell de Fortuny, Mihail Lazar Mioc, Joan Gómez‐Junyent, Gerard Solanellas Manzanares, Joan Felipe Soler, Daniel Pérez‐Prieto

**Affiliations:** ^1^ Department of Orthopedics and Traumatology “Pius Brinzeu” Emergency Clinical County Hospital Timisoara Romania; ^2^ Doctoral School of Medicine “Victor Babes” University of Medicine and Pharmacy Timisoara Romania; ^3^ Orthopedic Department, Hospital del Mar Universitat Pompeu Fabra Barcelona Spain; ^4^ Department XV Orthopedics and Traumatology “Victor Babes” University of Medicine and Pharmacy Timisoara Romania; ^5^ Department of Infectious Diseases, Hospital del Mar, Infectious Pathology and Antimicrobial Research Group (IPAR) Universitat Pompeu Fabra Barcelona Spain; ^6^ Center for Biomedical Research in Infectious Diseases Network (CIBERINFEC) Instituto de Salud Carlos III Madrid Spain; ^7^ Hospital Dexeus Barcelona Spain

**Keywords:** infection eradication, metaphyseal cone, periprosthetic joint infection, porous tritanium, revision knee arthroplasty

## Abstract

**Purpose:**

Metaphyseal cones improve fixation and address bone loss in revision total knee arthroplasty (rTKA), but their use in septic revision remains controversial because of theoretical concerns about biofilm formation on porous surfaces, and evidence confined to the septic setting is scarce. We aimed to describe infection eradication, implant survival and functional outcomes and the metaphyseal microbiological environment after cone implantation in septic rTKA.

**Methods:**

This multicentre retrospective single‐arm cohort study included consecutive patients undergoing septic rTKA with at least one porous tritanium metaphyseal cone at two centres between January 2016 and December 2023. Periprosthetic joint infection was diagnosed by the European Bone and Joint Infection Society (EBJIS) 2021 criteria. In one‐stage revisions, an additional metaphyseal bone sample was obtained during reaming for cone implantation. The primary outcome was infection eradication at a minimum 24‐month follow‐up; infection‐free implant survival was estimated by Kaplan–Meier. Secondary outcomes were metaphyseal culture positivity in one‐stage patients and functional scores (Knee Society Score [KSS], Western Ontario and McMaster Universities Osteoarthritis Index [WOMAC], EuroQol five‐dimension five‐level questionnaire [EQ‐5D‐5L], EuroQol Visual Analogue Scale [EQ‐VAS]) at last follow‐up.

**Results:**

Thirty‐four patients (mean age 70.3 ± 8.3 years; 55.9% female) were included: 18 (52.9%) one‐stage and 16 (47.1%) two‐stage revisions. Median follow‐up was 44 months (interquartile range [IQR]: 35–50). Infection was eradicated in 31 patients (91.2%, 95% confidence interval [CI]: 75.1%–97.1%); all three failures occurred by 14 months. Metaphyseal cultures were positive in 12 of 18 one‐stage patients (66.7%, 95% CI: 43.7–83.7). Cones were removed in three patients. Median functional scores were KSS Knee 80, KSS Functional 60, WOMAC Physical Function 17 and EQ‐VAS 70.

**Conclusion:**

In this cohort, cone implantation in septic rTKA was associated with a high infection eradication rate and durable implant survival, with three cone removals. The 66.7% metaphyseal culture positivity in one‐stage patients is an exploratory finding that may reflect occult infection or sample contamination and cannot be generalised beyond this subgroup. These findings support cones as a reconstructive option in septic rTKA.

**Level of Evidence:**

Level IV.

**Abbreviations**
AORI: Anderson Orthopaedic Research Institute
ASA: American Society of Anesthesiologists
BMI: body mass index
CCK: Condylar constrained knee
CFU/mL: colony‐forming units per millilitre
CI: confidence interval
EBJIS: European Bone and Joint Infection Society
EQ‐5D‐5L: EuroQol five‐dimension five‐level questionnaire
EQ‐VAS: EuroQol Visual Analogue Scale
IQR: interquartile range
KSS: knee society score
PJI: periprosthetic joint infection
PMN/HPF: polymorphonuclear leukocytes per high‐power field
rTKA: revision total knee arthroplasty
SD: standard deviation
STROBE: strengthening the reporting of observational studies in epidemiology
TKA: total knee arthroplasty
WOMAC: Western Ontario and McMaster Universities Osteoarthritis Index

## INTRODUCTION

Despite advances in surgical technique and perioperative care, treatment of infected total knee arthroplasty (TKA) still carries failure rates of 15%–25% within 2 years of septic revision [2, 8, 25, 27]. Bone loss, infection‐related osteolysis and aggressive debridement complicate reconstruction and threaten implant fixation [7, 18]. Porous metal metaphyseal cones address substantial bone defects and provide biological fixation through osseointegration, particularly for Anderson Orthopaedic Research Institute (AORI) type II and III defects [5, 9, 11, 12]. Their use in aseptic revision is well established, but their application in the septic setting remains controversial [16, 21]. The main concern is that porous surfaces may harbour bacterial colonisation and biofilm formation. Available evidence in the septic setting is limited and reports heterogeneous outcomes for infection eradication, implant survival and function [6, 24, 28]. Cone fixation is well documented in aseptic revision, whereas series confined to the septic setting are rare, largely because of the fear of implanting a large porous implant into a previously or currently infected field. To our knowledge, no previous study has cultured the metaphyseal bone removed during cone preparation.

We aimed to describe infection eradication, implant survival and functional outcomes in a consecutive single‐arm cohort of patients undergoing septic revision TKA with metaphyseal cone implantation and to characterise the microbiological findings at the metaphyseal interface in the subgroup undergoing one‐stage revision. Given the retrospective noncomparative design, the study cannot establish the comparative safety or effectiveness of cone implantation relative to alternative reconstructive strategies. We hypothesised that the reaming required to seat a cone results in additional metaphyseal debridement and explored whether this was associated with maintained infection control and acceptable clinical functional outcomes in this cohort.

## METHODS

This was a multicentre retrospective single‐arm cohort study with prospective data collection conducted at two arthroplasty referral centres. The study involved reviewing revision arthroplasty databases at both institutions to identify consecutive patients who underwent septic revision total knee arthroplasty (rTKA) with implantation of metaphyseal cones between January 2016 and December 2023. Preoperative radiographic evaluation was performed in all patients, including standardised views for bone defect classification according to the AORI system and full‐length weight‐bearing lower limb radiographs for surgical planning [9]. All patients were followed up through scheduled in‐person clinical visits at 1, 3, 6, 12 and 24 months postoperatively, and every 2 years thereafter.

Patients were included if they had a confirmed periprosthetic joint infection, diagnosed according to the EBJIS 2021 criteria and underwent septic rTKA with at least one metaphyseal cone implanted, either tibial, femoral or both. A minimum follow‐up of 24 months was required.

Patients were excluded if their clinical or microbiological records were incomplete, since this would have made it impossible to evaluate the primary outcome. Patients who died before reaching the 24‐month mark were also excluded for the same reason. Patients requiring a higher constraint than a condylar‐constrained type (CCK) insert were excluded.

The study adheres to the strengthening the reporting of observational studies in epidemiology (STROBE) guidelines for cohort studies.

### Surgical technique and perioperative protocol

All procedures were performed through a midline skin incision and with a medial parapatellar approach. Thorough synovectomy, debridement of all necrotic and fibrous tissue and removal of all components and cement were performed.

A standardised diagnostic protocol was used, consisting of:

– sampling of five periprosthetic tissue specimens
– sonication of the removed implant
– prolonged incubation of synovial and periprosthetic tissues
– histopathological analysis for acute and chronic inflammation.

Metaphyseal cone implantation was generally indicated in AORI defects II and III. However, the final decision to implant a cone was made intraoperatively based on the assessment of metaphyseal bone integrity and fixation stability. AORI classification was performed by two senior arthroplasty surgeons in consensus. Individual cases with AORI I defects therefore received a cone when the surgeon judged that additional metaphyseal support was required. Subsequently, pulsatile lavage was performed, first with 3 L of a 0.05% chlorhexidine solution and then with 3 L of saline. Following this, the metaphyseal cavity was prepared using a high‐speed burr and sequential cylindrical reamers until cortical contact was achieved (Figure 1b); the reamed tissue was sent for additional microbiological analysis. The porous tritanium cone (Stryker Corp.) was then impacted to the appropriate depth, rotation and orientation using size‐specific instrumentation (Figures 1a and 2b). The Stryker Triathlon revision system with a CCK insert and cemented diaphyseal stem extensions was used in all cases (Figure 2a). The metaphyseal cone was press‐fit into the reamed cavity for biological fixation; antibiotic‐loaded cement (Simplex Tobramycin; Stryker Corp.) was applied at the cone‐implant (stem) interface, not at the cone‐bone itself. The same cone system and the same debridement, pulsatile lavage and cement were used at both participating centres during the study. The choice between one‐stage and two‐stage revision was determined by a multidisciplinary team according to internationally established clinical criteria (EBJIS 2021) [17]. Patients started empiric intravenous antibiotics with induction until culture results were available; afterwards, antibiotic therapy was targeted according to the multidisciplinary team's decision. Immediate full weight‐bearing was permitted in all patients. Physiotherapy commenced on the first postoperative day, at four sessions per week for 2 months. All patients were assessed with lateral and frontal x‐rays at follow‐up visits. Radiographic loosening was defined by the presence of progressive radiolucent lines, radiolucencies >2 mm, cone migration or subsidence compared with previous radiographs.

**Figure 1:**
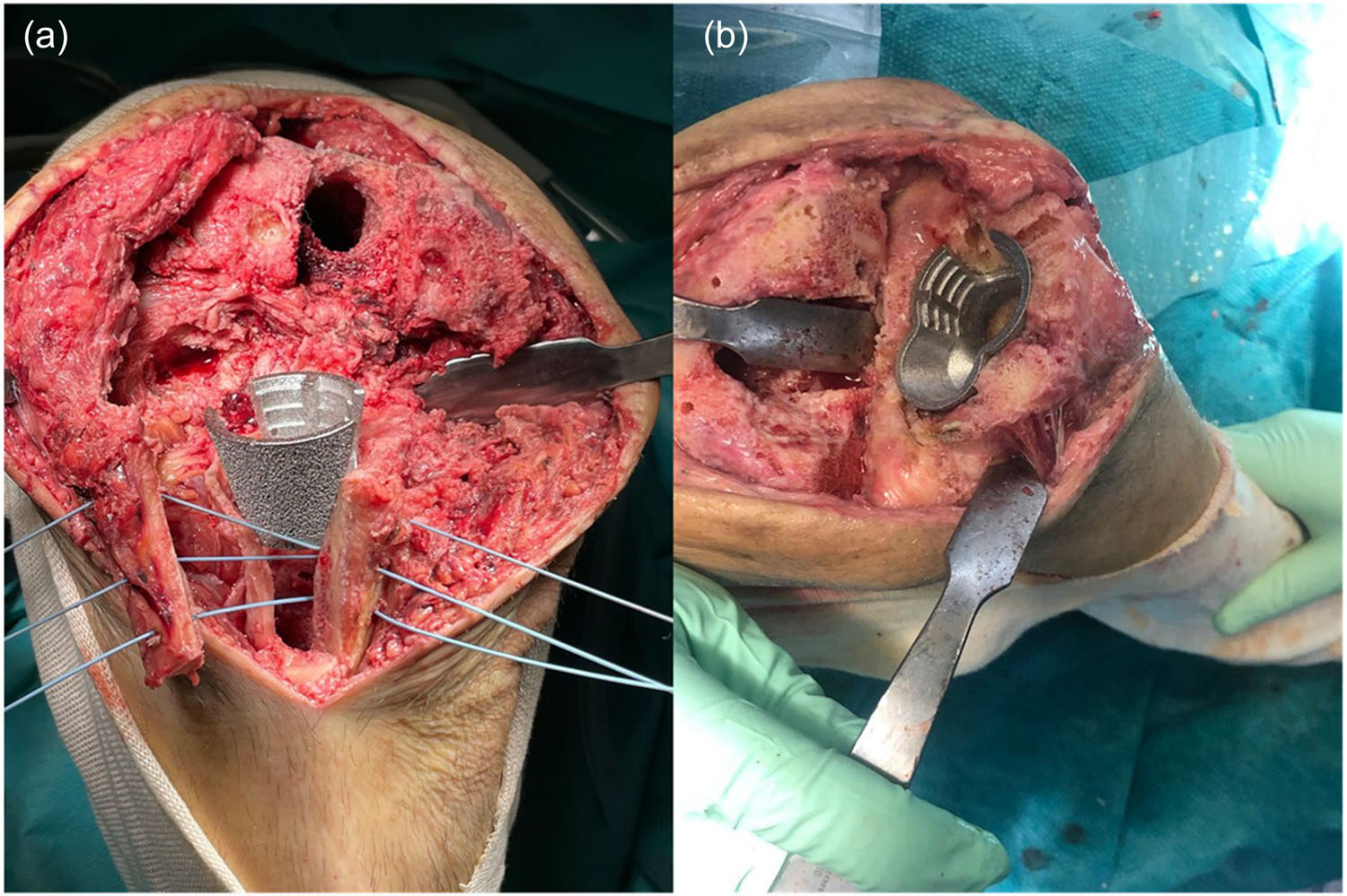
(a) Intraoperative axial view of the tibial plateau following cone implantation. The porous tritanium tibial cone is seated centrally within the metaphyseal cavity. Significant tibial bone loss consistent with an AORI T2 defect is visible, with the residual metaphyseal cortex providing peripheral support. (b) Intraoperative view of the tibial metaphysis during preparation for an asymmetric cone implantation. AORI, Anderson Orthopaedic Research Institute.

**Figure 2:**
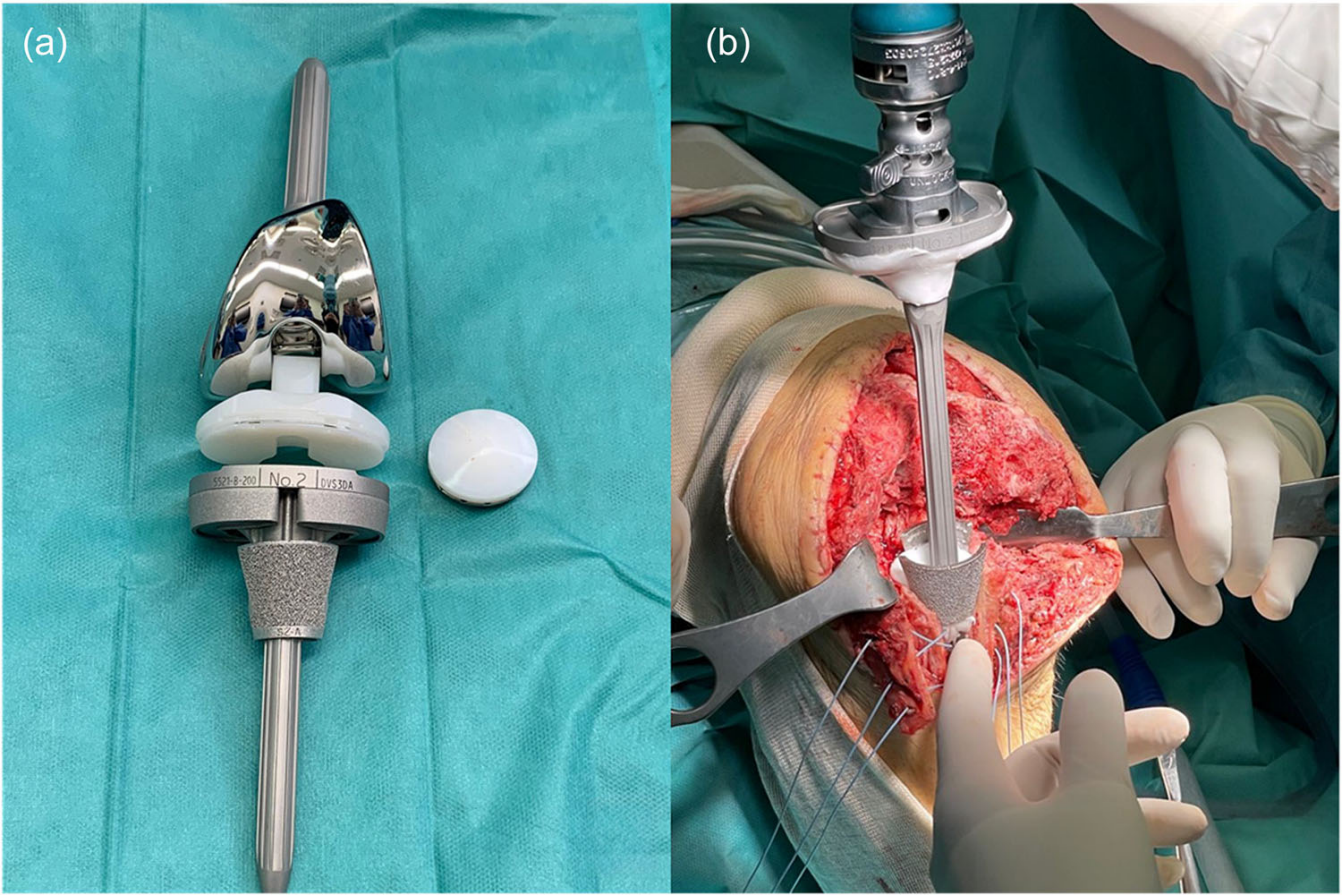
(a) The Stryker Triathlon revision system used in all cases: femoral component with cemented stem extension, condylar‐constrained (CCK) polyethylene insert, tibial baseplate, porous tritanium tibial metaphyseal cone and tibial stem extension. Patellar button shown separately. (b) Intraoperative tibial implant seating and cementation of the cone and stemmed tibial plate.

### Microbiological and histopathological analysis

A standardised microbiological protocol was applied in all cases. At the index revision surgery, a minimum of five periprosthetic tissue samples were collected from representative anatomical sites (synovial membrane, posterior capsule, tibial bone‐cement interface, femoral bone‐cement interface and canal tissue). Removed components were subjected to sonication using a validated protocol; a colony count of 50 or more colony‐forming units per millilitre (CFU/mL) was considered the positivity threshold. All samples were cultured under both aerobic and anaerobic conditions for a minimum of 14 days [3]. One additional tissue sample per patient was submitted for histopathological examination; the presence of more than five polymorphonuclear leucocytes per high‐power field (> 5 PMN/HPF) was considered indicative of active infection. In one‐stage revisions, one additional metaphyseal bone sample was obtained after pulsatile lavage and after reamer preparation of the metaphyseal cavity, immediately before cone impaction using a dedicated sterile instrument kit to limit cross‐contamination from the joint field. The same positivity threshold was applied to this sample as to periprosthetic tissue specimens. For these patients, the organism isolated from the metaphysis was compared with the one isolated from the periprosthetic samples of the same patient. This sampling strategy was specifically designed to characterise the microbiological environment at the metaphyseal cone interface. Metaphyseal tissue cultures were not obtained in two‐stage patients at the index procedure, because cone implantation in this group occurs at the second‐stage implantation, after an antibiotic‐free interval and standard reassessment of infection control, rather than at the time of initial debridement. At second‐stage reimplantation, intraoperative tissue samples, intraoperative aspiration and aspirate periprosthetic cultures were obtained according to standard protocol, but no dedicated metaphyseal sample was taken.

### Data collection and outcomes

Data collected include demographic and clinical variables: age, sex, body mass index (BMI), American Society of Anesthesiologists (ASA) physical status classification (reported as I–II vs. III–IV for this analysis), predisposition for immunocompromised state (chronic use of corticosteroids, Type 2 Diabetes Mellitus, Chronic Kidney Disease stages 3–4, liver cirrhosis), history of prerevision implant, type of revision strategy (one‐stage vs. two‐stage), location of the metaphyseal cone (tibia, femur or both), AORI classification of bone defects in each case (femur or tibia separately), isolated microorganism(s), duration of antibiotic treatment and follow‐up period.

The primary outcome was represented by infection eradication in patients who had at least 24 months of follow‐up. Eradication was defined as: absence of clinical, radiographic and laboratory findings of infection, no need for antibiotics to suppress infection, no further surgeries required for a septic cause, and no death related to infection [16]. The need for further antibiotic therapy was considered treatment failure regardless of whether reintervention was performed.

Secondary outcomes were the frequency of positive metaphyseal tissue cultures at the time of index revision surgery (only for patients with one‐stage) and patient reported outcome measurements at the last follow‐up (Knee Society Score [KSS]; knee and functional subscores), Western Ontario and McMaster Universities Osteoarthritis Index (WOMAC); pain, stiffness and physical function subscales), the EQ‐5D‐5L [EuroQol‐5D five‐level questionnaire; mobility, self‐care, usual activities, pain/discomfort, anxiety/depression] and the EQ‐VAS [EQ Visual Analogue Scale]). Secondary outcomes were analysed in 33 of 34 patients; one patient was excluded due to a preexisting L4–L5 radiculopathy with motor deficit affecting the operative limb that limited the evaluation of surgical outcomes.

### Data analysis

Statistical analysis was conducted in R (version 4.3). No formal a priori sample‐size calculation was performed; this was a retrospective cohort study including all consecutive eligible cases at both centres of the study period. Continuous variables that were found to be normally distributed according to the Shapiro–Wilk test were summarised as mean ± SD, whereas median and interquartile range (IQR) were used for nonnormally distributed variables. Categorical variables were described as counts and percentages. 95% confidence intervals for binomial proportions were calculated using the Wilson score method. Infection‐free implant survival was defined as a primary endpoint and estimated with the Kaplan–Meier method. Time‐to‐event was measured from the day of the index revision surgery until either confirmed failure or the last clinical follow‐up without failure occurred. Deceased patients were censored at their last known visit. Long‐term mortality surveillance beyond the scheduled follow‐up visits was not a defined study endpoint; the reported deaths were identified because they occurred among patients still attending scheduled follow‐up, and the cause of death was available from clinical records for each. These deaths were censored at the last known visit rather than modelled as a competing risk for infection‐free survival. In terms of exploratory analysis, survival curves for one‐stage and two‐stage revision groups were compared using the log‐rank test. The association between follow‐up duration and each functional outcome score was tested using the Spearman rank correlation coefficient. A level of statistical significance of *α* = 0.05 was considered throughout.

### Ethical approval

This study was approved by the local Institutional Review Board and conducted in accordance with the Declaration of Helsinki.

## RESULTS

Of 36 patients initially identified, two were excluded for failure to reach the minimum 24‐month follow‐up due to death from causes unrelated to periprosthetic joint infection (PJI) before this threshold. Thirty‐four patients met the inclusion criteria and represented the final cohort (Figure 3). Baseline demographic and surgical characteristics are summarised in Table 1.

**Figure 3:**
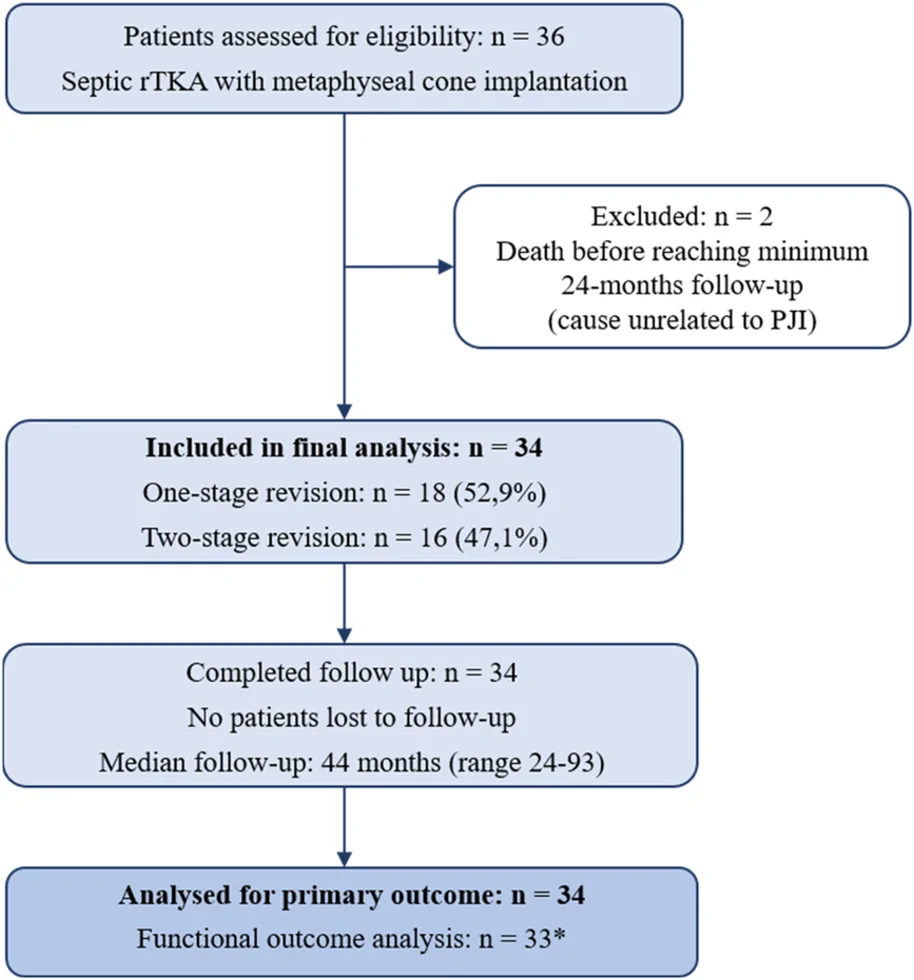
STROBE flow diagram. *One patient excluded from functional analyses due to L4–L5 radiculopathy with motor deficit affecting the operative limb. PJI, periprosthetic joint infection; rTKA, revision total knee arthroplasty; STROBE, strengthening the reporting of observational studies in epidemiology.

**Table 1:** Patients demographics.

Variable	Total cohort (*n* = 34)
Age (years)	70.3 ± 8.3
Sex—Female	19 (55.9%)
BMI (kg/m^2^)	28.9 ± 4.2
ASA physical status	
ASA I–II	21 (61.8%)
ASA III–IV	13 (38.2%)
Any immunosuppression	10 (29.4%)
Prerevision implant—primary TKA	31 (91.2%)
Prerevision implant—prior revision	3 (8.8%)
Revision strategy	
1‐stage	18 (52.9%)
2‐stage	16 (47.1%)
Cone location	
Tibial only	28 (82.4%)
Tibial + femoral	5 (14.7%)
Femoral only	1 (2.9%)
AORI classification—femur	
I	23 (67.6%)
II	2 (5.9%)
II	8 (23.5%)
AORI classification—tibia	
I	3 (8.8%)
II	19 (55.9%)
III	12 (35.3%)
Metaphyseal tissue culture (1‐stage only, *n* = 18)	
Positive	12 (66.7%)
Negative	6 (33.3%)
Follow‐up duration (months)	44 (35–50)

*Note*: Data presented as mean ± SD, median (IQR) or *n* (%). One patient had no femoral bone defect requiring classification (AORI) femur, *n* = 33/34. Abbreviations: AORI, Anderson Orthopaedic Research Institute; ASA, American Society of Anesthesiologists; BMI, body mass index; TKA, total knee arthroplasty.

Revision was performed as a one‐stage procedure in 18 patients (52.9%) and two‐stage in 16 (47.1%). Median follow‐up was 44 months (IQR: 35–50; range 24–93).

The most frequently identified causative microorganisms were *Staphylococcus epidermidis* (*n* = 12, 35.3%; 95% CI: 21.5%–52.1%), *Staphylococcus aureus* (*n* = 6, 17.6%; 95% CI 8.3%–33.5%) and *Streptococcus mitis* (*n* = 3, 8.8%; 95% 3.0%–23.0%). Among the 18 patients treated with a one‐stage strategy, metaphyseal tissue cultures were obtained in all cases, yielding positive results in 12 (66.7%; 95% CI: 43.75%–83.7%). Among the 12 one‐stage patients with positive metaphyseal cultures, 11 (91.7%; 95% CI: 64.6%–98.5%) achieved infection eradication; the single failure in the one‐stage subgroup occurred in a metaphyseal culture‐positive patient. All six patients with negative metaphyseal cultures achieved infection eradication. In all 12 patients with a positive metaphyseal culture, both cultures matched the same microorganism. No patient had a positive metaphyseal culture with negative periprosthetic cultures. Second‐stage microbiological assessment at reimplantation was positive in 2 of 16 patients (12.5%; 95%CI: 3.5%–36.0%). Of note, one patient with positive aerobic and anaerobic soft tissue cultures at the second stage subsequently experienced treatment failure requiring arthrodesis at 14 months postrevision. We found no radiologically loose implants.

Infection was eradicated in 31 of 34 patients. The Kaplan–Meier estimate for infection‐free implant survival was 91.2% at 14 months (95%CI: 75.1%–97.1%) and remained stable thereafter (Figure 4); median survival was not reached. All three treatment failures (8.8%; 95%CI: 3.0%–23.0%) occurred within the first 14 months: one patient (two‐stage group) was placed on indefinite suppressive antibiotic therapy at 6 months for treatment failure according to EBJIS 2021 criteria; the second patient (one‐stage group) had reinfection after 7 months and was managed with a two‐stage procedure; the third patient (two‐stage group) had persistent infection despite reimplantation and required knee arthrodesis at 14 months. The patient who underwent arthrodesis was the only two‐stage case with microbiologically confirmed infection persistence at reimplantation. An exploratory comparison of survival curves between one‐stage (*n* = 18) and two‐stage (*n* = 16) groups was performed but was uninterpretable given the small number of events (log‐rank *p* = 0.483; Figure 5); this comparison should not be read as evidence of equivalence between the two strategies. Four deaths were identified during follow‐up, none related to PJI (two cardiovascular, one traumatic brain injury, one SARS‐CoV‐2 complication). All four patients who died during follow‐up had already reached the minimum 24‐month threshold at the time of death and were retained in the final analytic cohort, in contrast to the two patients who died before reaching 24 months and were excluded.

**Figure 4:**
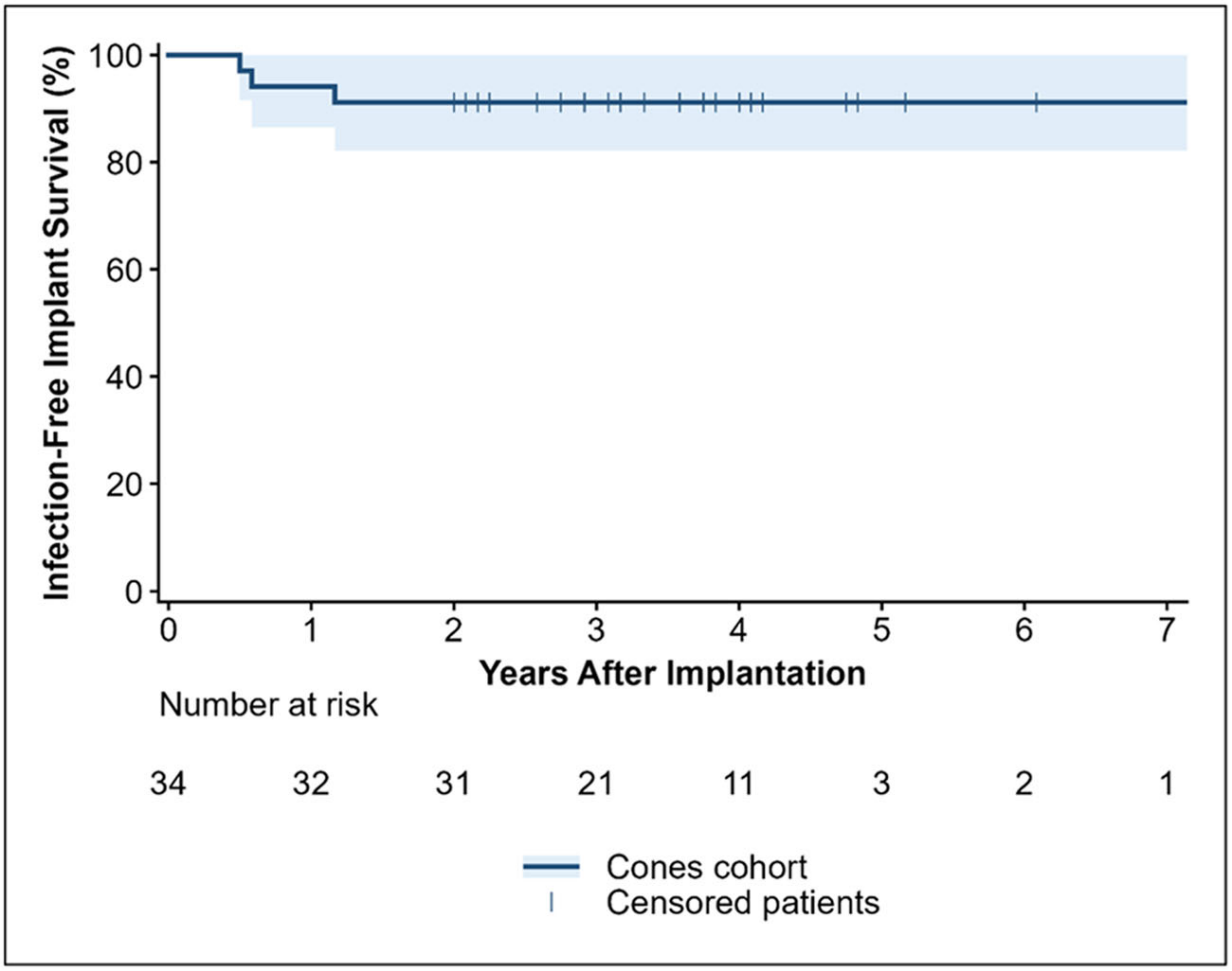
Kaplan–Meier curve for infection‐free implant survival.

**Figure 5:**
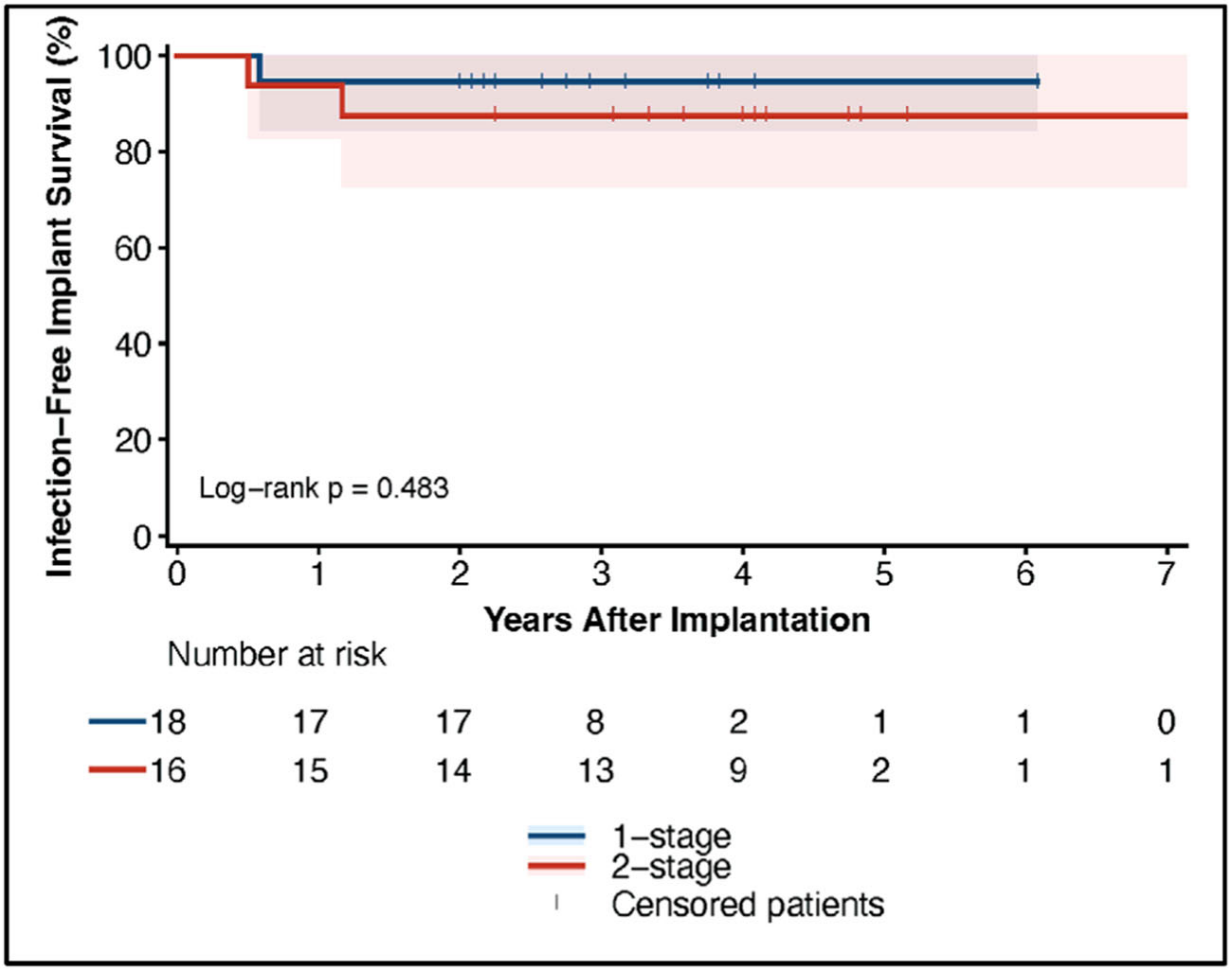
Kaplan–Meier curves for infection‐free implant survival by revision strategy.

Six patients (17.6%; 95% CI: 8.3%–33.5%) underwent at least one reoperation on the index knee following revision surgery. Two reoperations were directly attributable to treatment failure: conversion to two‐stage revision (*n* = 1) and knee arthrodesis (*n* = 1). The remaining four reoperations were performed for reasons unrelated to infection recurrence: polyethylene exchange for instability (*n* = 1), femoral component revision for stem‐tip pain (*n* = 1), haematoma debridement (*n* = 1) and arthrolysis for stiffness (*n* = 1). Cones were removed in three knees: at conversion to two‐stage revision, at arthrodesis and at femoral component revision for stem‐tip pain. No cone was removed for aseptic loosening.

### Functional outcomes and follow‐up correlation

Functional outcomes at final follow‐up are summarised in Table 2. Two patients showed marked discordance between EQ‐VAS of 90 and the lowest objective scores in the cohort; one assessed after arthrodesis for treatment failure, one with extensive contralateral knee pathology. As an exploratory analysis, Spearman rank correlation detected no statistically significant association between follow‐up duration and any functional outcome score (Table 3); given the modest sample size, this analysis is underpowered to detect small associations and should be read descriptively rather than as evidence of no relationship.

**Table 2:** Primary and secondary outcomes.

Variable	Total cohort (*n* = 34)
Infection eradication (primary outcome)*	31 (91.2%)
KSS knee subscore (0–100)**	80 (49–94)
KSS functional subscore (0–100)**	60 (13–80)
WOMAC pain**	4 (2–9)
WOMAC stiffness**	1 (0–2)
WOMAC physical function**	17 (7–40)
EQ‐VAS global health**	70 (50–80)
EQ‐5D mobility**	2 (1–4)
EQ‐5D self‐care**	1 (1–3)
EQ‐5D usual activities**	1 (1–4)
EQ‐5D pain/discomfort**	2 (1–3)
EQ‐5D anxiety/depression**	1 (1–3)

*Note*: Data presented as *n* (%) for the primary outcome and median (IQR) for functional outcome measures. (*) Primary outcome; 95% Confidence Interval, 75.1%–97.1%. (**) Assessed in *n* = 33 patients; one patient was excluded due to a preexisting neurological condition.

Abbreviations: EQ‐5D‐5L, EuroQol five‐dimension five‐level questionnaire; EQ‐VAS, EuroQol Visual Analogue Scale; IQR, interquartile range; KSS, Knee Society Score; WOMAC, Western Ontario and McMaster Universities Osteoarthritis Index.

**Table 3:** Correlation between follow‐up duration and functional outcomes (*n* = 33).

Variable	Spearman *ρ*	*p*‐value	Pearson *r* (sensitivity)	*p*‐Value
KSS knee subscore	−0.318	0.071	−0.347	0.048
KSS functional subscore	−0.08	0.657	‐	‐
WOMAC pain	+0.223	0.212	‐	‐
WOMAC stiffness	+0.106	0.558	‐	‐
WOMAC physical function	+0.274	0.123	+0.346	0.048
EQ‐VAS	+0.253	0.155	‐	‐

*Note*: Pearson *r* reported as sensitivity analysis only; not considered a primary finding given confirmed nonnormal distribution of both variables (Shapiro–Wilk *p* = 0.002) and the disproportionate influence of the arthrodesis data point at 93 months.

Abbreviations: EQ‐VAS, EuroQol Visual Analogue Scale; KSS, Knee Society Score; WOMAC, Western Ontario and McMaster Universities Osteoarthritis Index.

## DISCUSSION

In this cohort of septic revision TKA, metaphyseal cone implantation was associated with an infection eradication rate of 91.2% (95% CI: 75.1%–97.1%) at a median follow‐up of 44 months, close to the 15%–25% 2‐year failure rates reported in the literature regardless of reconstruction technique [4, 13, 15, 22]. All failures occurred within the first 14 months, after which the Kaplan–Meier curve plateaued.

The theoretical concern that porous metal in a contaminated field would harbour biofilm and perpetuate infection [10, 16, 21] is not supported by our findings. Porous tantalum, a metallurgically distinct material from the porous titanium used in this study, has been reported to have intrinsic antibacterial properties in some publications [23, 26], however, a systematic review and meta‐analysis of 881 knees found no significant difference in PJI incidence between tantalum‐augmented and nontantalum implants (OR: 0.52, 95% CI: 0.13–2.0) [19], and whether any material‐specific antibacterial effect extends to the porous titanium (Tritanium) construct used here remains unproven. Our results should therefore be read as evidence against a clinically important biofilm risk from porous titanium itself, rather than as support for an antibacterial property of the material.

Metaphyseal cultures were positive in 12 of 18 one‐stage patients (66.7%; 95%CI: 43.7–83.7), showing that bacteria remained in metaphyseal bone after debridement and lavage. In every case, the metaphyseal isolate matched the periprosthetic organism, which argues against contamination. Metaphyseal positivity carried no prognostic weight, as 11 of 12 positive and all six negative patients were cured. Two limits apply: organisms were matched by species, not by strain, so chance agreement cannot be excluded for *S. epidermidis*; and all positive patients also had positive joint cultures, so the data show persistence of the known organism, not a separate source.

Septic‐only cone series are rare. Ohlmeier et al. reported a 7.7% revision rate (2 septic, 2 aseptic) among 52 revision knees with porous tibial cones, including 17 septic cases, at a mean follow‐up of 22 months [21]. The series are not directly comparable as they contain different outcome measures. One‐stage exchange for PJI unrelated to cone reconstruction has separately achieved around 90% infection control in other implant systems [20], consistent with our findings. Reliable cone and sleeve fixation at 5 years has been shown in mostly aseptic cohorts [7, 12, 14, 24]. In our cohort, no cones were removed for aseptic loosening, and no retained cones showed radiographic loosening; the three cones removed were explanted during reoperation for persistent infection. This is consistent with preserved osseointegration in the septic setting [7, 12], although the number of events is small.

Functional outcomes were heterogeneous but acceptable for this complex population: satisfactory objective knee function (median KSS Knee 80) and expected residual limitation in an elderly cohort (KSS Functional 60), with scores stable over follow‐up. Two patients showed marked discordance between high EQ‐VAS (90) and low objective scores, possibly reflecting the disability paradox. This is a recognised phenomenon in which patients with chronic functional limitation adapt their internal reference standard and report preserved well‐being despite objective impairment [1].

This study has several limitations. The retrospective design and absence of a control group limit causal inference. Treatment allocation was clinical rather than randomised. The 66.7% (95% CI: 43.7–83.7) metaphyseal culture positivity rate rests on the 18 one‐stage patients sampled and cannot be generalised. The 34‐patient sample yielded only three failure events, precluding multivariable analysis and producing a wide primary‐outcome confidence interval. The absence of preoperative functional scores prevents quantification of functional improvement after revision. Two patients who died from unrelated causes before the 24‐month threshold were excluded, introducing survivor bias. Similarly, the four deaths occurring after inclusion were censored rather than treated as a competing risk for infection‐free survival; although none were temporally or clinically related to infection, this approach cannot fully exclude informative censoring, and larger future cohorts should consider a competing‐risk framework. In particular, the exploratory comparison between one‐stage and two‐stage revision strategies is underpowered, with only three failure events distributed across both groups. This comparison should not be interpreted as evidence of equivalence between the two strategies. Statements regarding cone implantation into an actively infected field apply specifically to the 18 one‐stage patients. Nonetheless, this multicentre series provides one of the few datasets addressing metaphyseal cone implantation in the septic setting.

Despite these limitations, this multicenter series represents the most focused dataset available on metaphyseal cone implantation in active PJI and offers a meaningful contribution to an area where evidence remains limited.

## CONCLUSION

Porous tritanium metaphyseal cone implantation was associated with an infection eradication rate of 91.2% (95% CI: 75.1%–97.1%) with durable implant survival in septic revision TKA. Metaphyseal cultures were positive in 66.7% (95% CI: 43.7%–83.7%) of one‐stage patients, an exploratory observation indicating that the causative organism may persist in metaphyseal bone after debridement. These findings support cone implantation as a viable option for TKA PJI.

## AUTHOR CONTRIBUTIONS

**Andrea Pasquini**: Methodology; formal analysis; data curation; writing—original draft; writing—review and editing. **Lucas Martorell de Fortuny**: Investigation; Data curation; writing—review and editing. **Mihail Lazar Mioc**: Formal analysis; writing—original draft; writing—review and editing. **Joan Gómez‐Junyent**: Conceptualisation; investigation; writing—review and editing. **Gerard Solanellas Manzanares**: Investigation; writing—review and editing. **Joan Felipe Soler**: writing—review and editing. **Daniel Pérez‐Prieto**: Conceptualisation; methodology; supervision; writing—original draft; writing—review and editing. All authors read and approved the final manuscript.

## FUNDING INFORMATION

Societat Catalana de Malalties Infeccioses i Microbiologia Clinica.

## CONFLICT OF INTEREST STATEMENT

The authors declare no conflicts of interest.

## ETHICS STATEMENT

This study was approved by the local Institutional Review Board (Comité Ética de Investigación con Medicamentos (CEIm)—Ethics Committee protocol no. 2020/9122/I) and conducted in accordance with the Declaration of Helsinki. Informed consent was obtained from all patients in accordance with local regulations.

## Data Availability

The data that support the findings of this study are available from the corresponding author upon reasonable request.
